# Palmitoylation of the TPβ isoform of the human thromboxane A_2_ receptor. Modulation of G protein: Effector coupling and modes of receptor internalization

**DOI:** 10.1016/j.cellsig.2006.12.001

**Published:** 2007-05

**Authors:** Helen M. Reid, B. Therese Kinsella

**Affiliations:** School of Biomolecular and Biomedical Sciences, Conway Institute of Biomolecular and Biomedical Research, University College Dublin, Belfield, Dublin 4, Ireland

**Keywords:** AR, adrenergic receptor, C-tail, carboxyl-terminal tail, [Ca^2+^]_*i*_, intracellular calcium, eNOS, endothelial nitric oxide synthase, GFP, green fluorescent protein, GPCR, G protein-coupled receptor, HA, hemagglutinin, HEK, human embryonic kidney, 5-hydroxytryptamine (4a) receptor, 5-HT_4(a)_, IP, prostacyclin receptor, IP_3_, inositol 1, 4, 5-trisphosphate, PAGE, polyacrylamide gel electrophoresis, PK, protein kinase, PL, phospholipase, TP, TXA_2_ receptor, TXA_2_, thromboxane A_2_, Thromboxane A_2_ receptor, Receptor internalization, Palmitoylation

## Abstract

Palmitoylation is a prevalent feature amongst G protein-coupled receptors. In this study we sought to establish whether the TPα and TPβ isoforms of the human prostanoid thromboxane (TX) A_2_ receptor (TP) are palmitoylated and to assess the functional consequences thereof. Consistent with the presence of three cysteines within its unique carboxyl-terminal domain, metabolic labelling and site-directed mutagenesis confirmed that TPβ is palmitoylated at Cys^347^ and, to a lesser extent, at Cys^373,377^ whereas TPα is not palmitoylated. Impairment of palmitoylation did not affect TPβ expression or its ligand affinity. Conversely, agonist-induced [Ca^2+^]_*i*_ mobilization by TPβ^C347S^ and the non-palmitoylated TPβ^C347,373,377S^, but not by TPβ^C373S^ or TPβ^C373,377S^, was significantly reduced relative to the wild type TPβ suggesting that palmitoylation at Cys^347^ is specifically required for efficient Gq/phospholipase Cβ effector coupling. Furthermore, palmitoylation at Cys^373,377^ is critical for TPβ internalization with TPβ^C373S^, TPβ^C373,377S^ and TPβ^C347,373,377S^ failing to undergo either agonist-induced or temperature-dependent tonic internalization. On the other hand, whilst TPβ^C347S^ underwent reduced agonist-induced internalization, it underwent tonic internalization to a similar extent as TPβ. The deficiency in agonist-induced internalization by TPβ^C347S^, but not by TPβ^C373,377^ nor TPβ^C347,373,377S^, was overcome by over-expression of either β-arrestin1 or β-arrestin2. Taken together, data herein suggest that whilst palmitoylation of TPβ at Cys^373,377^ is critical for both agonist- and tonic-induced internalization, palmitoylation at Cys^347^ has a role in determining which pathway is followed, be it by the β-arrestin-dependent agonist-induced pathway or by the β-arrestin-independent tonic internalization pathway.

## 1 Introduction

The covalent addition of lipid moieties to proteins is widely recognized as an important regulatory mechanism in eukaryotic cells contributing to protein:membrane and/or protein:protein interactions [1,2]. Common lipidations include the co-translational attachment of myristate acid via a stable amide linkage to amino (N)-terminal glycine residues, post-translational attachment of farnesyl or geranylgeranyl isoprenoids to carboxy (C)-terminal cysteine(s) via thioether bond(s) and post-translational addition of palmitate or other fatty acyl group to cysteine(s) via labile thioester linkages [1–5]. The latter, generally referred to as palmitoylation but more accurately termed acyl thioesterification or S-acylation, is the least understood despite being the most common lipid modification [6]. Unlike myristoylation or isoprenylation, palmitoylation is a dynamic modification with proteins constantly undergoing enzymatic cycles of palmitoylation and depalmitoylation, events that are thought to regulate not only membrane/protein interactions but also protein function [1,6]. This has been supported with the recent identification of candidate mammalian protein:S-acyl-transferases (PATs) and S-acylprotein thioesterases (APTs) that catalyze such palmitoylations and depalmitoylations, respectively [5,6].

A diverse range of palmitoylated proteins have been identified, most notably amongst proteins participating in intracellular signalling including H- and N-Ras, endothelial nitric oxide synthase, growth cone-associated protein 43, SRC and several other protein tyrosine kinases ([4,5] and references therein). Palmitoylation is also a feature of heterotrimeric Gα subunits and of their downstream effectors, including Gαs, Gα_q_ and adenylyl cyclase [1,4,5] and is a common modification of numerous G protein-coupled receptors (GPCRs). In fact, it is estimated that up to 80% of GPCRs contain at least one palmitoylable cysteine typically located some 10–14 amino acid residues downstream from transmembrane (TM) 7 within their carboxyl-terminal (C)-tail domain [7]. Rhodopsin, the first GPCR to be confirmed to be palmitoylated, undergoes palmitoylation at Cys^322^ and Cys^323^ within its C-tail domain and it was proposed that interaction of the hydrophobic palmitoyl moieties with the lipid bilayer of the plasma membrane may result in the formation of a fourth intracellular loop within its GPCR structure [8]. Numerous other GPCRs have since been shown to be palmitoylated within their C-tail domains including the α_2A_- and β_2_-adrenergic receptors [9,10], the dopamine D_1_ receptor [11,12], the 5-hydroxytryptamine (4a) receptor [13], the muscarinic acetylcholine receptor [14], the prostacyclin receptor [15] and the vasopressin V_1A_ and V_2_ receptors [16,17]. Through these and many other specific examples, it is evident that while palmitoylation may potentially affect diverse aspects of GPCR function and regulation including processing and targeting, protein turnover, G protein coupling, phosphorylation and desensitization, sequestration and/or internalization [1–5], it is also apparent that such effects are generally receptor specific, requiring investigation at the individual GPCR level.

The prostanoid thromboxane (TX)A_2_ plays a critical role in vascular hemostasis regulating platelet activation status and vascular tone [18]. In humans, but not in non-primates, TXA_2_ signals through two distinct isoforms of the TXA_2_ receptor (TP) termed TPα and TPβ [18–21]. As members of the GPCR superfamily, TPα and TPβ are identical for their first N-terminal 328 amino acids and diverge exclusively within their carboxyl-terminal (C)-tail domains [18–22]. While TPα and TPβ arise by differential splicing [20], they display distinct patterns of mRNA and protein expression [23,24] and, more recently, it has been established that they are under the transcriptional regulation of distinct promoters within the single human TP gene [25–28]. Moreover, whilst both TPα and TPβ exhibit identical ligand binding and Gq-dependent activation of phospholipase (PL) Cβ, their primary effector [20,29–31], they oppositely regulate adenylyl cyclase through Gs and Gi, respectively [32] and TPα, but not TPβ, can couple to Gh/tissue transglutaminase activation [33].

Hence, it is evident that TPα and TPβ display differences in their patterns of expression and in their modes of intracellular signalling, suggesting that they may have distinct physiologic roles [22]. Consistent with this hypothesis, there is an abundance of emerging evidence suggesting that the greatest differences between TPα and TPβ lies in their modes of desensitization/regulation post-receptor signalling, through events that are largely regulated through sequences or functional determinants within their respective unique C-tail domains. For example, while both TPs undergo agonist-induced phosphorylation [24,29], TPβ, but not TPα, is subject to both agonist-induced and tonic internalization [34,35]. Agonist-induced TPβ internalization appears to be dynamin-, GRK2/3- and β-arrestin-dependent [34,35], requiring an active or dynamic actin cytoskeleton [36] and may also involve direct interaction between Rab 11 and the C-tail domain of TPβ [37]. On the other hand, tonic internalization of TPβ is both GRK- and β-arrestin-independent but dynamin-dependent and is also dependent on the presence of an intact internalization motif within its C-tail domain [34,35]. In studies investigating cross-talk between TXA_2_ and other prostanoids, it was established that signalling by TPα, but not TPβ, is subject to prostacyclin- and prostaglandin (PG)D_2_-mediated desensitization involving direct PKA phosphorylation of TPα at Ser^329^ within its unique C-tail domain [30,38]. Consistent with this, TPα, but not the TPβ, is also a target for nitric oxide (NO)-induced desensitization that occurs through a mechanism involving its direct PKG phosphorylation at Ser^331^ within its unique C-tail domain [39]. These latter studies point to an essential role for TPα in prostacyclin- and NO-regulated vascular hemostasis and point to a redundant or an, as yet, unidentified role for TPβ in this essential physiologic process [22,30,38,39]. Moreover, collectively, these data also indicate that TPα and TPβ are subject to distinct modes of regulation, intracellular signalling and expression and that such differences are largely determined by sequences within their unique C-tail domains.

Another notable difference between the TP isoforms, the significance of which has yet to be investigated, is the presence of multiple cysteine (Cys) residues within the unique C-tail of TPβ at Cys^347^, Cys^373^ and Cys^377^, while the C-tail domain of the TPα is entirely devoid of such residues. The presence of Cys residues within the unique C-tail domain of TPβ raises the possibility that they may be palmitoylated and that such modification(s) may contribute to the functional differences between the two TP isoforms. Hence, the aim of the current study was to investigate whether TPβ is subject to palmitoylation and to examine the functional consequences thereof. Our studies confirm that TPβ, but not TPα, is palmitoylated at Cys^347^ and, to a lesser extent, at Cys^373/377^, a modification that not only regulates agonist-induced second messenger signalling but also differentially influences agonist- and tonic-induced internalization of TPβ.

## 2 Experimental procedures

### 2.1 Materials

U46619 and SQ29,548 were obtained from Cayman Chemical Company. G418 sulfate and FURA2/AM were from Calbiochem. [^3^H]SQ29,548 (50.4 Ci/mmol) was obtained from DuPont NEN. Rat monoclonal 3F10 anti-HA-horse radish peroxidase-conjugated antibody (25 μg/ml) was obtained from Roche. Mouse monoclonal anti-hemagglutinin (HA)-101R antibody (1 mg/ml) was obtained from Eurogentec; goat anti-β-arrestin1 (K-16), goat anti-β-arrestin2 (N-16), horse radish peroxidase (HRP)-conjugated goat anti-mouse (400 μg/ml), HRP-conjugated mouse anti-goat (400 μg/ml) and HRP-conjugated goat anti-rabbit (400 μg/ml) secondary antibodies were from Santa Cruz; goat anti-mouse FITC-conjugated antibody, propidium iodide, protein G sepharose 4B Fast Flow (∼ 50% v/v slurry) were obtained from Sigma. AlexaFluor594 goat anti-mouse antibody (2 mg/ml) was from Molecular Probes. [9, 10-^3^H] Palmitic acid (60 Ci/mmol) was purchased from TOCRIS.

### 2.2 Site-directed mutagenesis of TPβ to generate TPβ^C347S^, TPβ^C373S^, TPβ^C373,377S^ and TPβ^C347,373,377S^

The plasmids pHM6:TPα, pHM6:TPβ and pHM6:hIP encoding HA epitope-tagged forms of TPα, TPβ and human prostacyclin receptor (IP), respectively, were previously described [30,40]. pRK5:β-arrestin1, pcDNA3:β-arrestin2, pEGFPN3:β-arrestin1 and pEGFPN1:β-arrestin2 encoding wild type and green fluorescent protein (GFP)-tagged forms of β-arrestin1 and β-arrestin2 have been described [41–43]. pCMV:Gα_q_, encoding Gα_q_, has been previously described [40,44].

Conversion of Cys^347^ to Ser^347^ of TPβ to generate pHM6:TPβ^C347S^ was achieved using pHM6:TPβ as template and the sense/antisense primer pair (5′ G ATC TCG GCT CAC ***TCC*** AAC CTC CGC CTC). Conversion of Cys^373^ to Ser^373^ to generate pHM6:TPβ^C373S^ was achieved using pHM6:TPβ as template and the sense/antisense primer pair (5′ GTA AGC CAC ***TCC*** GCC CGG CCT TGC). Conversion of Cys^373,377^ to Ser^373,377^ to generate pHM6:TPβ^C373,377S^ was performed using pHM6:TPβ as template and the sense/antisense primer pair (5′ GTA AGC CAC ***TCC*** GCC CGG CCT ***TCC*** ATG CTC TTT G). The plasmid pHM6:TPβ^C347,373,377S^ was generated using pHM6:TPβ^C347S^ as template and sense/antisense primer pair (5′ GTA AGC CAC ***TCC*** GCC CGG CCT ***TCC*** ATG CTC TTT G). For each primer pair, sequences shown correspond to the sense primer and the identity of the mutator codon is in boldface italics. All site-directed mutagenesis was performed using QuikChange™ (Stratagene) site-directed mutagenesis and all mutations were validated by DNA sequence analysis.

### 2.3 Cell culture and transfections

Human embryonic kidney (HEK) 293 cells were obtained from the American Type Culture Collection and were grown in minimal essential medium (MEM) containing 10% foetal bovine serum (FBS).

Routinely, approximately 48 h prior to transfection cells were plated at a density of 2 × 10^6^ cells/10 cm culture dish in 8 ml media. Thereafter, cells were transiently transfected with 10 μg of pADVA [45] and 25 μg of pCMV- or pHM-based vectors using the calcium phosphate/DNA co-precipitation procedure, as previously described [44]. For transient transfections, cells were harvested 48 h after transfection. To create stable cell lines, HEK 293 cells were transfected with 10 μg *Sca*1 linearized pADVA plus 25 μg *Sca*1 linearized pHM-based vectors. Forty-eight hours post-transfection, G418 (0.8 mg/ml) was added; individual G418 resistant colonies were selected after approximately 21 days and clonal cell lines were expanded and were assessed for TP expression by radioligand binding assay using the radioligand [^3^H]SQ29,548. In this way, HEK.TPβ^C347S^, HEK.TPβ^C373S^, HEK.TPβ^C373,377S^ and HEK.TPβ^C347,373,377S^ cell lines stably over-expressing HA-tagged forms of TPβ^C347S^, TPβ^C373S^, TPβ^C373,377S^ and TPβ^C347,373,377S^, respectively, were established. HEK.TPα, HEK.TPβ and HEK.hIP cells stably over-expressing HA-tagged form of TPα, TPβ and of the human IP, respectively, have been previously described [30,40].

### 2.4 Radioligand binding studies

TP radioligand binding assays were carried out at 30 °C for 30 min in the presence of 0–40 nM [^3^H]SQ29,548 for Scatchard analyses or in the presence of 20 nM [^3^H]SQ29,548 for saturation radioligand binding experiments as previously described [44]. Protein determinations were carried out using the Bradford assay.

### 2.5 Palmitoylation of the thromboxane receptor

Palmitoylation was carried out essentially as previously described [15]. Briefly, cells were plated 48 h in advance to achieve a density of approx. 3 × 10^5^ cells/10-cm dish (∼ 80% confluency) on the day of metabolic labelling. Cells were washed once in PBS and then metabolically labelled in serum-free MEM (1.5 ml) containing 1.5 mCi of [^3^H] palmitic acid (60 Ci/mmol). Following incubation for 2 h at 37 °C, the labelling reaction was stopped by washing the cells in ice-cold PBS. Thereafter, cells were lysed by the addition of 600 μl RIPA buffer (20 mM Tris–Cl, pH 8.0, 0.15 M NaCl, 1% Triton X-100, 1% SDS, 1% deoxycholate, 10 mM EDTA, pH 8.0, 1 mM PMSF, 2 mM 1,10-phenanthroline, 10 μg/ml aprotinin, 10 μg/ml antipain, 1 μg/ml leupeptin, 10 μg/ml benzamidine) and were disrupted by passing through needles of decreasing bore size. The lysate was centrifuged at 13,000 rpm for 5 min and the supernatant (600–700 μg) was subjected to immunoprecipitation using the anti-HA 101R antibody (1:300 dilution) followed by the addition of 10 μl protein G Sepharose 4B (50% v/v slurry) and further incubation at room temperature for 1 h, as described [15]. Immunoprecipitates were then resuspended in SDS-PAGE sample buffer (25 mM Tris–Cl, pH 8.0, 1% SDS, 5% glycerol, 0.0013% (w/v) Bromophenol Blue, 1% β-Mercaptoethanol), incubated at room temperature for 15 min, and then resolved by 8% SDS-PAGE. After electroblotting onto PVDF membrane, the blots were soaked in Amplify (Amersham) for 30 min followed by fluorography using Kodak X-Omat XAR film for 60 days at − 70 °C. Thereafter, following fluorographic exposure, PVDF membranes were screened by immunoblot analysis using the anti-HA 3F10 peroxidase-conjugated antibody followed by chemiluminescence detection.

### 2.6 Calcium measurements

Measurements of agonist-induced intracellular calcium ([Ca^2+^]_*i*_) mobilization in response to the TXA_2_ mimetic U46619 (1 μM) were carried out in FURA2/AM preloaded HEK.TPβ, HEK.TPβ^C347S^, HEK.TPβ^C373S^, HEK.TPβ^C373,377S^ and HEK.TPβ^C347,373,377S^ cells that had been transiently co-transfected with pCMV:Gα_q_ and pADVA some 48 h previously, essentially as described [44]. The agonist U46619, in ethanol, was diluted 1:1000 in the vehicle HBSSHB (modified Ca^2+^/Mg^2+^-free Hank's buffered salt solution, containing 10 mM HEPES, pH 7.67, 0.1% bovine serum albumin) and the agonist in vehicle (20 μl) or, as controls, the vehicle alone (20 μl) were added to the 2 ml cells (0.8 × 10^6^ cells/ml) to achieve the desired working concentration. It was established that the vehicle had no effect on [Ca^2+^]_*i*_ mobilization by either TP isoform and had no effect on experimental data. The ratio of the fluorescence at 340 nm to 380 nm is a measure of [Ca^2+^]_*i*_ [46], assuming a *K*_d_ of 225 nM Ca^2+^ for FURA2/AM. The results presented in the figures are representative data from at least three/four independent experiments and are plotted as changes in intracellular Ca^2+^ mobilized (Δ[Ca^2+^]_*i*_ (nM)) as a function of time (seconds, s) following agonist stimulation.

### 2.7 Internalization of TP receptors

Cells were seeded at a density of 5 × 10^4^ cells/ml per well in 1 ml of MEM, 10% FBS media into 24-well plates, which had been pre-coated with 0.001% poly-l-lysine. The cells were then incubated for 48 h at 37 °C prior to experiments. To assess agonist-induced internalization of TPβ or its mutated derivatives, the media was changed to serum-free Dulbecco's modified essential medium and thereafter, cells were treated with 1 μM U46619 for 0, 1, 2 and 4 h at 37 °C. Cells were washed twice in ice-cold PBS prior to fixation in 3.7% paraformaldehyde, 1× PBS, pH 7.4, for 15 min at room temperature. After washing the cells in 1× TBS (20 mM Tris–HCl, pH 7.4, 0.1 M NaCl), non-specific sites were blocked with Blocking Buffer (5% dried skimmed milk powder in TBS) and then cells were incubated with anti-HA 101R antibody (1:1000 in Blocking Buffer) for 1 h at room temperature. The antibody solution was removed and the cells were washed twice in TBS, prior to incubation with goat anti-mouse HRP (1:1000) for 45 min at room temperature. Thereafter, the cells were washed trice in TBS and HA-tagged receptor expression was detected colorimetrically at 650 nm using the K-Blue substrate [47].

To assess tonic internalization, 48 h post-seeding, cells were washed in MEM, 10% FBS, prior to incubation in media containing mouse anti-HA 101R (1 in 1000 dilution), at 4 °C for 1 h. Thereafter, the cells were rinsed briefly in MEM to remove unbound antibody prior to incubating the cells at 4 °C and 37 °C for 2 h. Cells were washed twice in ice-cold PBS prior to fixation in 3.7% paraformaldehyde, 1× PBS, pH 7.4, for 15 min at room temperature. After washing the cells twice in 1× TBS (20 mM Tris–HCl, pH 7.4, 0.1 M NaCl), non-specific sites were blocked with Blocking Buffer followed by incubation with the secondary goat anti-mouse HRP (1:1000) for 45 min at room temperature. Detection of the HA-tagged receptors was via colorimetric K-Blue substrate (Neogen Corp), as previously described [47].

### 2.8 Immunofluorescence and confocal microscopy

Cells were seeded onto poly-l-lysine pre-treated coverslips in 6-well plates to achieve 60–70% confluency following 48 h incubation at 37 °C. Thereafter, the media was changed to MEM, 10% FBS and cells were incubated with anti-HA 101R (1 in 1000 dilution) at 4 °C for 1 h. Thereafter, unbound antibody was removed by washing twice with media. To investigate agonist-induced internalization, cells were washed once with serum-free MEM prior to incubation with 1 μM U46619 for 0, 2 and 4 h at 37 °C. To examine tonic internalization, following labelling with anti-HA 101R (1 in 1000 dilution) at 4 °C for 1 h, cells were washed briefly in MEM to remove unbound antibody prior to incubation at 4 °C and 37 °C for 2 h in MEM, 10% FBS.

After the appropriate incubation, cells were washed twice in ice-cold PBS prior to fixation in 3.7% paraformaldehyde, 1× PBS, pH 7.4, for 15 min at room temperature and thereafter washed three times with PBS. Cells to be permeabilized were then incubated with 0.2% Triton X-100 in PBS for 10 min on ice followed by washing in 1× TBS. Non-specific sites were blocked by incubating cells with Blocking Buffer. Thereafter, receptors that had been previously labelled with the anti-HA 101R antibody were labelled with the secondary goat anti-mouse FITC-conjugated antibody solution. Cells were washed 3 times in 1× TBS, prior to counterstaining with propidium iodide (1 μg/ml, in H_2_O). Excess propidium iodide was washed away with H_2_O prior to mounting coverslips in Vectashield (Vector Laboratories) mounting medium. Slides were then imaged using Carl Zeiss Lazer Scanning System LSM510 using Zeiss LSM Imaging software.

### 2.9 β-arrestin1 and β-arrestin2 co-localization with TPβ receptors

HEK 293 cells (10-cm dishes, 60–70% confluent) were transiently co-transfected with pHM6-based plasmid encoding HA-tagged TPβ (365 ng), or its mutated variants, along with either pEGFPN3:β-arrestin1 or pEGFPN1:β-arrestin2, encoding green fluorescent protein (GFP)-tagged forms of the β-arrestin1/2 (365 ng), in the presence of pADVA (270 ng) using the Effectene (Qiagen) transfection reagent, as per manufacturer's instructions. Some 24 h post-transfection, cells were replated onto fibronectin (50 μg/ml, 2 μg/cm^2^) pre-coated glass coverslips (22 mm diameter) in 6-well plates and incubated for a further 48 h. Thereafter, cells were incubated with anti-HA 101R (1:1000 dilution in MEM, 10% FBS) at 4 °C for 1 h. Unbound antibody was removed by washing twice with media prior to incubating cells with 1 μM U46619 (prepared in media) for 30 min at 37 °C. Thereafter, cells were fixed in 3.7% paraformaldehyde, 1× PBS, pH 7.4, for 15 min at room temperature. Following washing (3 times with 1× PBS), cells were permeabilized by incubation on ice for 10 min in 0.2% Triton X-100 in PBS. Following washing twice in 1× TBS (20 mM Tris–HCl, pH 7.4, 0.1 M NaCl) and further incubation for 30 min with Blocking Buffer (5% dried skimmed milk powder in 1× TBS), HA-tagged receptors, previously labelled with the anti-HA 101R antibody, were visualized using the AlexaFluor594 goat anti-mouse IgG (1:1500) as secondary antibody. Cells were washed trice in TBS, prior to counterstaining with DAPI (1 μg/ml, in H_2_O). Excess DAPI was washed away with H_2_O prior to mounting coverslips in Vectashield mounting medium. A Zeiss fluorescence microscope coupled with AxioVision software (version 4.4) was used for acquiring multichannel, Z-stack images (19 slices, 0.125 μM apart) with filters appropriate for enhanced GFP and AlexaFluor 594 fluorescence. Subsequently, the multichannel, Z-stack images were processed by AxioVision Deconvolution Analysis, with enabled multichannel and Z-stack correction.

### 2.10 Data analyses

Radioligand binding data was analysed using GraphPad Prism V3.0 to determine the *K*_d_ and *B*_max_ values. Statistical analyses were carried out using the unpaired Student's *t* test using the Statworks Analysis Package. *p*-Values of less than or equal to 0.05 were considered to indicate a statistically significant difference.

## 3 Results

### 3.1 Palmitoylation of the human TXA_2_ receptor, TP

**Fig. 1:**
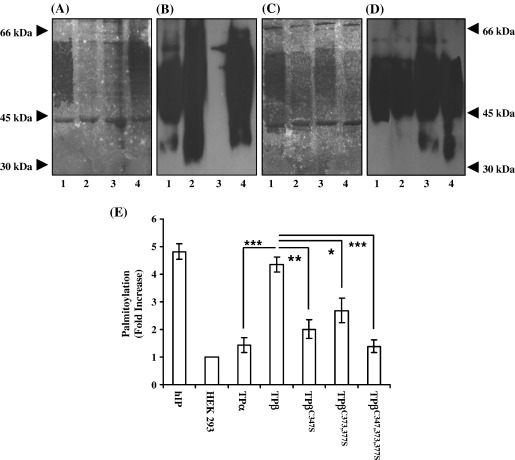
Analysis of palmitoylation in HEK.TPα and HEK.TPβ cells. Panels A and B, HEK.TPβ (lane 1), HEK.TPα (lane 2), HEK 293 (lane 3, negative control) and HEK.hIP (lane 4, positive control) cells were metabolically labelled for 2 h at 37 °C (Panel A). Panels C and D, HEK.TPβ (lane 1), HEK.TPβ^C347S^ (lane 2), HEK.TPβ^C373,377S^ cells (lane 3) and HEK.TPβ^C347,373,377S^ (lane 4) cells were metabolically labelled for 2 h at 37 °C (Panel C). Thereafter, the HA-tagged receptors were immunoprecipitated with anti-HA 101R and resolved by SDS-PAGE followed by electroblotting onto PVDF membrane. The blots (A and C) were soaked in Amplify prior to fluorography for 60–90 days at − 70 °C. Following fluorographic exposure, PDVF membranes in A and C were screened by immunoblot analysis using anti-HA 3F10 peroxidase-conjugated antibody followed by chemiluminescent detection to obtain Panels B and D, respectively. The positions of the molecular weight markers (kDa) are indicated to the left and right of Panels A and D, respectively. Panel E, the level of palmitoylation in HEK.hIP, HEK.TPα, HEK.TPβ, HEKHA.TPβ^C347S^, HEK.TPβ^C373,377S^ and HEK.TPβ^C347,373,377S^ cells relative to basal levels, in HEK 293 cells, was determined by Phosphorimage analysis. Data is presented as mean fold increase in palmitoylation over basal levels ± S.E.M. and are expressed in arbitrary units. The data are representative of three independent experiments. The asterisks indicate that palmitoylation levels of TPα, TPβ^C347S^, TPβ^C373,377S^ and TPβ^C347,373,377S^ cells were significantly lower than that of TPβ where ^⁎^, *p* < 0.05; ^⁎⁎^, *p* < 0.01; ^⁎⁎⁎^*p* < 0.005.

To investigate whether the human thromboxane (TX) A_2_ receptor (TP) isoforms are palmitoylated, human embryonic kidney (HEK) 293 cells stably over-expressing hemagglutinin (HA) epitope-tagged forms of TPα and TPβ were metabolically labelled with [^3^H] palmitic acid. As a control, metabolic labelling was also investigated in HEK.hIP cells stably over-expressing HA-tagged human (h) prostacyclin receptor (hIP), a GPCR previously confirmed to be palmitoylated [15]. Metabolic labelling and palmitoylation of both TPβ and the hIP was readily detected as evidenced by the presence of broad radiolabelled bands between 45 and 66 kDa markers in the immunoprecipitates from HEK.TPβ and HEK.hIP cells (Fig. 1A, lanes 1 and 4, respectively). In contrast, no metabolic labelling was associated with the immunoprecipitates from HEK.TPα cells or from control HEK 293 cells (Fig. 1A, lanes 2 and 3, respectively) confirming that, unlike that of TPβ, the TPα isoform does not undergo palmitoylation. Confirmation of equivalent expression and efficient recovery of the hIP, TPα and TPβ receptors in their respective immunoprecipitates were obtained by subsequent screening of the PVDF membrane by immunoblot analysis (Fig. 1B, lanes 1, 2 and 4, respectively) which also clearly indicated that failure to detect palmitoylation of TPα was not due to failure of its immunoprecipitation.

From Phosphorimage analysis, it was evident that both TPβ and the hIP were efficiently palmitoylated showing near equivalent 4.36- and 4.82-fold increases in palmitoylation relative to basal levels in HEK 293 cells, respectively (Fig. 1E). The efficient metabolic labelling of the hIP is consistent with the fact that this receptor undergoes palmitoylation at multiple Cys residues within its C-tail domain [15]. In the case of TPβ, it is notable that there are three cysteine residues within its unique C-terminal tail domain which may potentially be palmitoylated, possibly accounting for its near equivalent metabolic labelling relative to that of the hIP (Fig. 1A and E). Thus, to investigate the possible involvement of one or more of those Cys residues in palmitoylation and the implications thereof for TPβ function, site-directed mutagenesis was used to mutate Cys^347^, Cys^373^ and Cys^377^ to Ser residues, either individually and collectively, to generate TPβ^C347S^, TPβ^C373S^, TPβ^C373,377S^ and TPβ^C347,373,377S^. HEK 293 cell stably over-expressing TPβ^C347S^, TPβ^C373S^, TPβ^C373,377S^ and TPβ^C347,373,377S^ were generated and initially characterised by saturation radioligand binding analysis using the selective TP antagonist SQ29,548. HEK.TPβ^C347S^, HEK.TPβ^C373S^, HEK.TPβ^C373,377S^ and HEK.TPβ^C347,373,377S^ cells were confirmed to display similar ligand binding characteristics (*K*_d_ and *B*_max_) relative to HEK.TPβ cells and the mutations *per se* had no affect on the receptor affinity (Table 1). It should be noted that despite repeated attempts, it was not possible to generate stable cell lines over-expressing TPβ^C377S^.

To investigate whether Cys^347^ and/or Cys^373,377^ are actual targets for TPβ palmitoylation, HEK.TPβ^C347S^, HEK.TPβ^C373,377S^ and HEK.TPβ^C347,373,377S^ cells were metabolically labelled with [^3^H] palmitic acid. Palmitoylation of TPβ and, to a lesser extent, of TPβ^C347S^ and TPβ^C373,377S^ was observed as evidenced by the broad radiolabelled band between 45 and 66 kDa in their respective immunoprecipitates (Fig. 1C, lanes 1–3, respectively). Phosphorimage analysis confirmed that while the levels of metabolic labelling of both TPβ^C347S^ and TPβ^C373,377S^ were significantly reduced relative to that of TPβ, palmitoylation of either mutated receptor was not actually abolished (compare 4.36-, 2.01- and 2.68-fold increase in palmitoylation for TPβ, TPβ^C347S^ and TPβ^C373,377S^, respectively; Fig. 1E). In contrast, the levels of metabolic labelling of TPβ^C347,373,377S^ (1.39-fold increase) were significantly reduced relative to TPβ (Fig. 1C, lanes 2 and 4, respectively; Fig. 1E) and, in fact, were found not to be significantly greater than basal levels in immunoprecipitates from HEK 293 cells. Confirmation of equivalent expression and recovery of the TPβ, TPβ^C347S^, TPβ^C373,377S^ and TPβ^C347,373,377S^ receptors in their respective immunoprecipitates was obtained by subsequent screening of the PVDF membrane by Western blot analysis (Fig. 1D, lanes 1–4, respectively) and confirmed that failure, or any reductions, in detecting palmitoylation was not due to failure of the immunoprecipitation itself.

Thus, in summary, it is evident that TPβ is palmitoylated at multiple Cys residues within its C-tail domain and that mutation of Cys^347^ and Cys^373,377^ leads to a significant reduction in metabolic labelling confirming that palmitoylation occurs at both Cys^347^ and Cys^373,377^. Moreover, we also observed a significant reduction in the level of metabolic labelling of TPβ^C373S^ confirming that Cys^373^ is specifically palmitoylated (data not shown). However, as it was not possible to generate stable cell lines over-expressing TPβ^C377S^, we cannot conclude with certainty whether Cys^377^ along with Cys^373^ is palmitoylated.

### 3.2 Effect of TPβ^C347S^, TPβ^C373S^, TPβ^C373,377S^ and TPβ^C347,373,377S^ on intracellular signalling

**Fig. 2:**
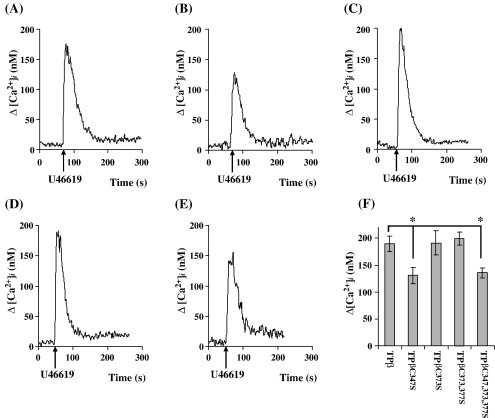
Analysis of U46619-mediated [Ca^2+^]_*i*_ mobilization. HEK.TPβ (Panel A), HEK.TPβ^C347S^ (Panel B), HEK.TPβ^C373S^ (Panel C), HEK.TPβ^C373,377S^ (Panel D) and HEK.TPβ^C347,373,377S^ (Panel E) cells, transiently co-transfected with pCMV5:Gα_q_ and preloaded with FURA2/AM, were stimulated with 1 μM U46619 where the ligand was added at times indicated by the arrows. The results are representative profiles from 4 independent experiments and are plotted as changes in intracellular Ca^2+^ mobilized (Δ[Ca^2+^]_*i*_, nM) as a function of time(s) following ligand stimulation. Data presented in Panel F is plotted as mean changes in Δ[Ca^2+^]_*i*_ mobilization (nM ± S.E.M.) for each cell line. The asterisk in Panel F indicates that U46619-mediated [Ca^2+^]_*i*_ mobilization by TPβ^C347S^ and TPβ^C347,373,377S^ was significantly lower than that of TPβ where ^⁎^, *p* < 0.05.

Numerous studies have indicated that the covalent attachment of palmitate has been implicated in the regulation of receptor G protein coupling and subsequent downstream signalling events [15,48]. To investigate whether palmitoylation has a role in Gq-coupled phospholipase (PL)Cβ activation by TPβ, we examined agonist-induced intracellular calcium ([Ca^2+^]_*i*_) mobilization by TPβ^C347S^, TPβ^C373S^, TPβ^C373,377S^ and TPβ^C347,373,377S^ in response to the TXA_2_ mimetic U46619. Consistent with previous reports [30], HEK.TPβ cells, transiently transfected with Gα_q_, showed efficient [Ca^2+^]_*i*_ mobilization in response to U46619 (Fig. 2A). Stimulation of TPβ^C373S^ or TPβ^C373,377S^ each showed efficient intracellular signalling and it was found that U46619-mediated [Ca^2+^]_*i*_ mobilization by either TPβ^C373S^ or TPβ^C373,377S^ was not significantly different to that of TPβ (Fig. 2C and D; *p* = 0.94 and 0.61, respectively). On the other hand, while TPβ^C347S^ did show a substantial signalling response (Fig. 2B), the level of [Ca^2+^]_*i*_ mobilization by TPβ^C347S^ was significantly reduced relative to that of TPβ, TPβ^C373S^ or TPβ^C373,377S^ (Fig. 2F; *p* = 0.02). Moreover, consistent with this, U46619-induced [Ca^+^]_*i*_ mobilization by TPβ^C347,373,377S^ was also impaired (Fig. 2E), showing significant reductions relative to that of TPβ, TPβ^C373S^ or TPβ^C373,377S^ (Fig. 2F; *p* = 0.01) whereas there was no significant difference in signalling between TPβ^C347S^ and TPβ^C347,373,377S^ (Fig. 2F; *p* = 0.79). Taken together, these data confirm that palmitoylation of TPβ plays a significant role in mediating its efficient coupling to Gq/PLCβ activation and, moreover, established that palmitoylation of Cys^347^ is specifically required for this regulation.

### 3.3 Effect of palmitoylation on agonist-induced internalization of TPβ

**Fig. 3:**
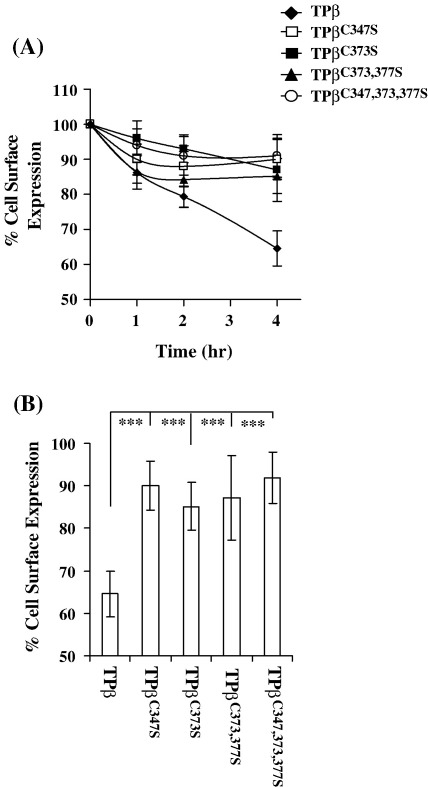
Effect of palmitoylation on agonist-induced internalization of TPβ. Panel A, HEK.TPβ, HEK.TPβ^C347S^, HEKHA.TPβ^C373S^, HEK.TPβ^C373,377S^ and HEK.TPβ^C347,373,377S^ cells were incubated with 1 μM U46619 at 37 °C for 0–4 h prior to fixation and immunolabelling with anti-HA 101R. Results are expressed as mean cell surface expression at each time point as a percentage of that at 0 h (% cell surface expression ± S.E.M., *n* = 3) as a function time (h). Panel B, mean cell surface expression following stimulation of cells with 1 μM U46619, 37 °C for 4 h expressed as a percentage of that at 0 h (% cell surface expression ± S.E.M., *n* = 3). The asterisks indicate that at 4 h, U46619-mediated loss of cell surface expression of TPβ^C347S^, TPβ^C373S^, TPβ^C373,377S^ and TPβ^C347,373,377S^ was significantly reduced relative to that of TPβ where ^⁎⁎⁎^, *p* < 0.0001.

TPβ, but not TPα, is widely reported to undergo significant agonist-induced and tonic internalization, albeit through distinct mechanisms [34,35]. Hence, to further investigate the functional consequences of TPβ palmitoylation, we also assessed and compared agonist-induced internalization of TPβ, TPβ^C347S^, TPβ^C373S^, TPβ^C373,377S^ and TPβ^C347,373,377S^, initially using an ELISA-based assay. Cells were treated with U46619 (1 μM) over a 4 h period, prior to fixation and immunolabelling with anti-HA 101R. Consistent with previous reports [34], TPβ underwent efficient agonist-induced internalization such that following 4 h exposure to U46619, its level of cell surface expression was reduced by approx. 40% (Fig. 3). On the other hand, mutation of any one of the Cys residues within the C-tail domain of TPβ, be it Cys^347^, Cys^373^ or Cys^377^ either singly or in combination, significantly impaired agonist-induced internalization (Fig. 3A). Specifically, following 4 h treatment with U46619, the level of cell surface expression of TPβ^C347S^, TPβ^C347,373,377S^, TPβ^C373S^ and TPβ^C373,377S^ had decreased by less than 10–15% (Fig. 3B).

**Fig. 4:**
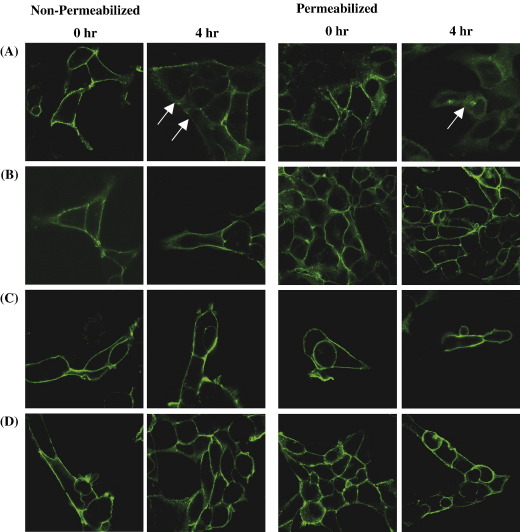
Confocal microscopy of agonist-induced internalization. HEK.TPβ (Panel A), HEK.TPβ^C347S^ (Panel B), HEK.TPβ^C373,377S^ (Panel C) and HEK.TPβ^C347,373,377S^ (Panel D) cells were with pre-labelled with anti-HA 101R, at 4 °C for 1 h, prior to stimulation with 1 μM U46619 at 37 °C for the times indicated. Thereafter, following fixation, surface expression of the HA-tagged receptors was detected, under both non-permeabilizing and permeabilizing conditions, by immunolabelling anti-mouse FITC-conjugated antibody. Images were captured using a Carl Zeiss Lazer Scanning System LSM510 and Zeiss LSM Imaging software. The arrows show agonist-induced TPβ internalization as indicated by loss of cell surface HA-tagged TPβ detected in non-permeabilized cells and increased detection of intracellular TPβ in permeabilized cells. The colour version of this figure is available online at www.sciencedirect.com.

Agonist-induced internalization of TPβ, TPβ^C347S^, TPβ^C373S^, TPβ^C373,377S^ and TPβ^C347,373,377S^ was also examined by immunofluorescence and confocal microscopy (Fig. 4). Owing to the fact that at any one time, even in the absence of agonist stimulation, a significant proportion of TPβ expression is found intracellularly, due at least in part to its tonic internalization and/or retention within the endoplasmic reticulum [35,49], to facilitate our studies cell surface receptors were initially immunolabelled with anti-HA 101R prior to exposure of cells with the agonist U46619. Immunolocalizations were then captured under non-permeabilizing and permeabilizing conditions at 0, 2 and 4 h (2 h data not shown throughout). In the absence of agonist (0 h), there was a strong detection of HA-tagged TPβ, TPβ^C347S^, TPβ^C373,377S^ and TPβ^C347,373,377S^ at their respective cell surfaces under both non-permeabilizing and permeabilizing conditions (Fig. 4, Panels A–D, 0 h). In the presence of U46619, cell surface expression of TPβ decreased with time, as indicated by reduced immunodetection in HEK.TPβ cells analysed in non-permeabilizing conditions, with a corresponding increase in TPβ internalization detected following permeabilization (Fig. 4A, 4 h). In permeabilized cells, TPβ expression was observed intracellularly at 4 h confirming that the HA-tagged TPβ had internalized from the cell surface into a cytoplasmic compartment. Indeed, there was a distinct punctuate staining within the perinuclear region, indicated by the arrow, appearing at the 4 h time point (Fig. 4A, permeabilized, 4 h). However, in contrast, the level of agonist-induced internalization of TPβ^C347S^ was significantly impaired relative to that of the wild type TPβ with no measurable decrease in surface expression and no increase in intracellular expression of TPβ^C347S^ detected under either non-permeabilizing or permeabilizing conditions (Fig. 4B). Moreover, U46619-stimulation did not result in a decrease in cell surface expression of TPβ^C373,377S^ and TPβ^C347,373,377S^ in cells analysed under non-pemeabilizing conditions, even following 4 h exposure to agonist (Fig. 4C and D, respectively) with no decrease in cell surface expression and no detection of internalized receptors when cells were analysed under permeabilizing conditions (Fig. 4C and D, 4 h). Similar results were observed for TPβ^C373S^ (data not shown).

Taken collectively, these data are consistent with previous studies confirming that TPβ is readily internalized in response to exposure to agonist [34]. However, in keeping with our ELISA-based assays, when palmitoylation of TPβ is disrupted, as in the case of either TPβ^C347S^, TPβ^C373S^, TPβ^C373,377S^ and/or TPβ^C347,373,377S^, the ability of TPβ to undergo agonist-induced internalization is impaired, showing no significant reduction in cell surface expression by either mutated variant.

### 3.4 Effect of palmitoylation on tonic internalization of TPβ

**Fig. 5:**
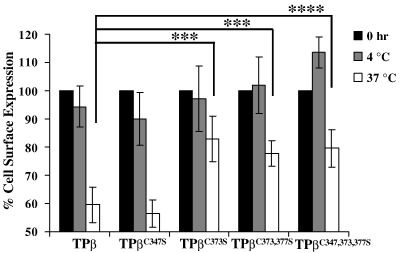
Effect of palmitoylation on tonic internalization. HEK.TPβ, HEK.TPβ^C347S^, HEK. TPβ^C373S^, HEK.TPβ^C373,377S^ and HEK.TPβ^C347,373,377S^ cells were incubated in the presence of anti-HA 101R for 1 h at 4 °C. Thereafter, following washing, cells were incubated at 4 °C or 37 °C for 2 h. Following fixation, cell surface expression of the HA-tagged receptors was detected using anti-mouse HRP conjugate antibody followed by colorimetric detection. Results are expressed as cell surface expression at 2 h as percentage of that at 0 h at the respective temperatures (% cell surface expression ± S.E.M., *n* = 3). The asterisks indicates that at 37 °C, tonic internalization of TPβ^C373S^, TPβ^C373,377S^ and TPβ^C347,373,377S^ was significantly reduced compared to that of TPβ where ^⁎^, *p* < 0.0001 and ^⁎⁎^, *p* < 0.0005.

As stated, TPβ, but not TPα, has also been reported to undergo tonic internalization through a temperature- and dynamin-dependent but GRK- and β-arrestin-independent manner [35]. Hence, due to the reduced ability of the palmitoylation deficient TPβ mutants to undergo agonist-induced internalization, we next sought to investigate the effect of palmitoylation on tonic internalization. Again, cell surface receptors in HEK 293 cells stably expressing TPβ, TPβ^C347S^, TPβ^C373S^, TPβ^C373,377S^ and TPβ^C347,373,377S^ were pre-labelled at 4 °C with anti-HA 101R prior to incubating the cells for 2 h at 4 °C and 37 °C, to evaluate tonic internalization initially using an ELISA-based assay. Consistent with previous reports [35], TPβ readily underwent tonic internalization as indicated by the 40% loss of cell surface expression of TPβ observed at 37 °C without any loss in such expression at 4 °C (Fig. 5). However, unlike that which occurred for its agonist-induced internalization, it was found that TPβ^C347S^ also underwent similar temperature-dependent tonic internalization to levels similar to that of the wild type TPβ. Specifically, it was found that cell surface expression of TPβ^C347S^ was reduced by approx. 40% at 37 °C and to a level that was not significantly different from that of the wild type TPβ (Fig. 5; *p* = 0.32). On the other hand, the level of tonic internalization of TPβ^C373S^, TPβ^C373,377S^ or TPβ^C347,373,377S^ was substantially impaired relative to TPβ (Fig. 5) or TPβ ^C347S^ (*p* < 0.0005) showing, on average, only a 15% loss of cell surface receptor expression following 2 h at 37 °C.

**Fig. 6:**
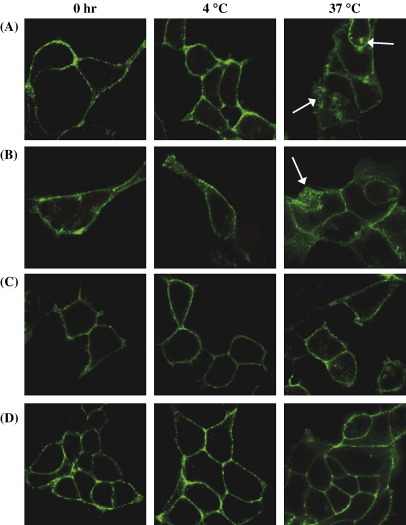
Confocal microscopy of tonic internalization. HEK.TPβ (Panel A), HEK.TPβ^C347S^ (Panel B), HEK.TPβ^C373,377S^ (Panel C) and HEK.TPβ^C347,373,377S^ (Panel D) cells were pre-labelled with anti-HA 101R at 4 °C for 1 h. Thereafter, following washing, cells were incubated for 2 h at 4 °C or 37 °C, followed by fixation in paraformaldehyde. Cell surface expression of the HA-tagged receptors was detected using anti-mouse FITC-conjugated antibody, under permeabilizing conditions. Images were captured using a Carl Zeiss Lazer Scanning System LSM510 and Zeiss LSM Imaging software. The arrows show temperature-induced tonic internalization of TPβ and TPβ^C347S^. The colour version of this figure is available online at www.sciencedirect.com.

To further investigate the influence of palmitoylation on internalization, we also monitored temperature-dependent tonic internalization of TPβ and its palmitoylation defective variants by confocal microscopy (Fig. 6). In brief, HEK 293 cell lines stably over-expressing HA-tagged TPβ, TPβ^C347S^, TPβ^C373,377S^ or TPβ^C347,373,377S^ were pre-labelled with anti-HA 101R at 4 °C; thereafter images were captured either directly (0 h), or following incubation for 2 h at 4 °C or 37 °C under non-permeabilizing (data not shown) and permeabilizing conditions (Fig. 6). At time 0 and following 2 h incubation at 4 °C, there was no evidence of internalized receptor for TPβ, TPβ^C347S^, TPβ^C373,377S^ or TPβ^C347,373,377S^ with all of the immunolabelled receptors showing cell surface expression. Following 2 h incubation at 37 °C, much of the expression of TPβ and TPβ^C347S^ was still found at the cell surface, albeit at a substantially reduced level, but in both cases a significant amount of the immunolabelled receptors was present intracellularly consistent with their temperature-dependent tonic internalization (Fig. 6A and B, 37 °C, indicated by arrows). As with the agonist-induced internalization of TPβ, the cytoplasmic staining of TPβ and TPβ^C347S^ exhibited a punctuate pattern in the perinuclear region. However, in the case of TPβ^C373,377S^ or TPβ^C347,373,377S^, there was little evidence of tonic internalization at 37 °C with both receptors being predominantly located at the cell surface (Fig. 6A and B, 37 °C). These data, particularly when taken in consideration with our ELISA-derived data, clearly indicates that the ability of TPβ^C373,377S^ and TPβ^C347,373,377S^ to undergo tonic internalization is severely impaired. Moreover, consistent with the ELISA data herein, the level of tonic internalization of TPβ^C373S^ following 2 h, or even more prolonged, incubation at 37 °C was severely impaired (data not shown).

Hence, in summary, whilst TPβ undergoes agonist-induced and tonic internalization, disruption of palmitoylation by mutating Cys^347^, Cys^373^ or Cys^377^, either singly or in combination, abolishes agonist-induced internalization. However, like TPβ, TPβ^C347S^ actually retains the ability to undergo tonic internalization while that of TPβ^C373S^, TPβ^C373,377S^ and TPβ^C347,373,377S^ is substantially impaired.

### 3.5 Effect of β-arrestin1 and -2 on TPβ^C347S^, TPβ^C373,377S^ and TPβ^C347,373,377S^ internalization

**Fig. 7:**
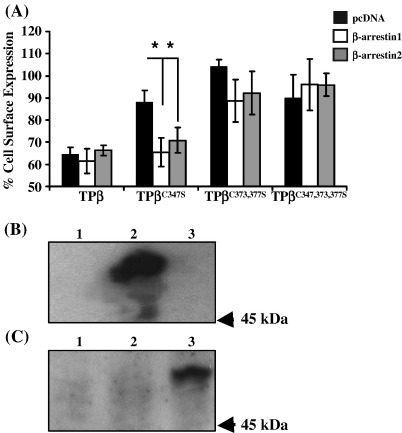
Effect of β-arrestin on agonist-induced internalization. Panel A, HEK.TPβ, HEK.TPβ^C347S^, HEK.TPβ^C373S^, HEK.TPβ^C373,377S^ and HEK.TPβ^C347,373,377S^ cells, transiently co-transfected with pRK5.β-arrestin1, pcDNA1.β-arrestin2 or, as a control, with the empty vector pcDNA were incubated with U46619 (1 μM) at 37 °C for 4 h, prior to fixation and immunolabelling with anti-HA 101R. Following fixation, cell surface expression of the HA-tagged receptors was detected using anti-mouse HRP-conjugated antibody followed by colorimetric detection. Results are expressed as mean cell surface expression at 4 h as a percentage of that at 0 h (% cell surface expression ± S.E.M., *n* = 3). The asterisk (^⁎^, *p* < 0.05) indicates that over-expression of β-arrestin1 and β-arrestin2 significantly reduced the level of cell surface expression of TPβ^C347S^ in the presence of U46619. Panels B and C, HEK.TPβ cells, transiently co-transfected with pRK5.β-arrestin1 (lane 2), pcDNA1.β-arrestin2 (lane 3) or, as a control, with the empty vector pcDNA (lane 1) were analysed by SDS-PAGE (50 μg whole cell protein analysed/lane) followed by Western blot analysis using anti-β-arrestin1 (K-16: Panel B) and anti-β-arrestin2 (N-16: Panel C). Data presented are representative immunoblots from three independent experiments. The relative position of the 45 kDa molecular size marker is indicated to the right of Panels B and C.

As stated, it is widely reported that agonist-induced internalization of TPβ shows a significant dependence on β-arrestin recruitment post-receptor activation and may, for example, be significantly reduced in the presence of dominant negative forms of either β-arrestin1 or β-arrestin2 [34]. Therefore, to further investigate the role of palmitoylation on TPβ internalization, we transiently transfected β-arrestin1 and β-arrestin2 into HEK 293 cells stably over-expressing TPβ and its variant TPβ^C347S^, TPβ^C373,377S^ and TPβ^C347,373,377S^ with the view to assessing their effect on agonist-mediated internalization. In the case of TPβ, consistent with previous reports [34], over-expression of the wild type forms of either β-arrestin1 or β-arrestin2 had no significant effect on the overall level of U46619-induced internalization following 4 h, as assessed by the ELISA-based internalization assay (Figs. 3 and 7A). However, whilst TPβ^C347S^ does not undergo significant agonist-induced internalization (Fig. 3), over-expression of β-arrestin1 and β-arrestin2 appeared to compensate and resulted in a 30% loss of cell surface receptor following exposure of cells to U46619 for 4 h (Fig. 7A, compare the level of agonist-induced internalization in HEK.TPβ^C347S^ cells transiently co-transfected with cDNA encoding β-arrestin1 and β-arrestin2 relative to the empty vector; *p* = 0.017 and *p* = 0.042, respectively). On the other hand, consistent with previous data (Figs. 3 and 4), TPβ^C373,377S^ and TPβ^C347,373,377S^ did not undergo agonist-induced internalization and over-expression of either β-arrestin1 and β-arrestin2 had no significant effect on the overall level of cell surface expression or internalization by either receptor type (Fig. 7A, compare pcDNA to β-arrestin1 and -2, *p* > 0.2 and *p* > 0.3, respectively). Over-expression of β-arrestin1 and β-arrestin2 was confirmed in HEK.TPβ cells by Western blot analysis using anti-β-arrestin1 and anti-β-arrestin2 (Fig. 7B and C, respectively).

**Fig. 8:**
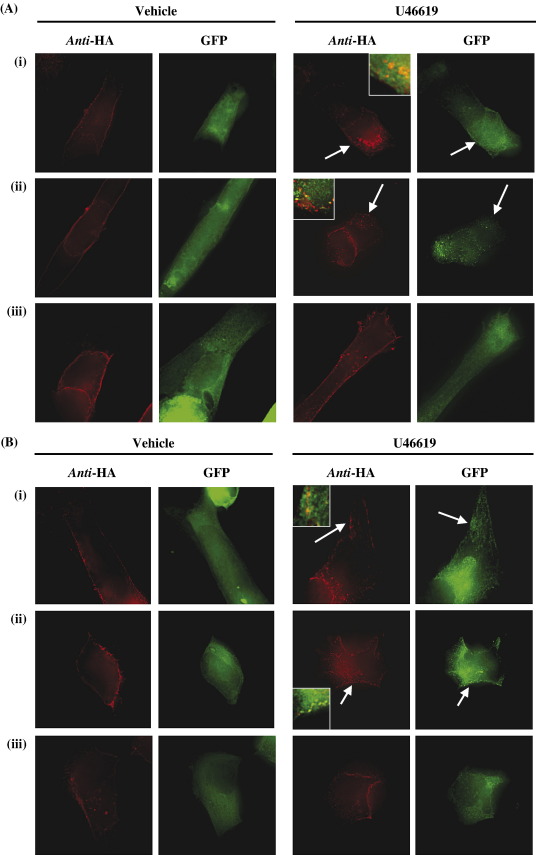
Effect of palmitoylation on TPβ and β-arrestin co-localization. Panels A and B, HEK 293 cells transiently co-transfected with (i) pHM6.TPβ, (ii) TPβ^C347S^ and (iii) TPβ^C347,373,377S^ along with pEGFPN3:β-arrestin1 (Panel A) or pEGFPN1:β-arrestin2 (Panel B) were immunolabelled with anti-HA 101R for 1 h at 4 °C. Thereafter, cells incubated with vehicle or U46619 (1 μM) for 30 min at 37 °C. Following fixation and permeabilization, cell surface HA-tagged TPs were labelled with the AlexaFluor 594 anti-mouse IgG. Images were captured using the Zeiss Fluorescence microscope with AxioVision software (version 4.4) and processed by AxioVision Deconvolution analysis. The arrows show agonist-induced β-arrestin1 and β-arrestin2 translocation and TPβ internalization while the insets to the panels show the co-localization of the β-arrestins with the TPβ receptors. The colour version of this figure is available online at www.sciencedirect.com.

To further assess the role of the β-arrestins in agonist-induced internalization of TPβ and its palmitoylation defective variants, HEK 293 cells were transiently co-transfected with respective plasmids encoding HA-tagged TPβ or its variants TPβ^C347S^, TPβ^C373S^ (data not shown), TPβ^C373,377S^ (data not shown) and TPβ^C347,373,377S^ along with GFP-tagged forms of either β-arrestin1 and β-arrestin2. HA-tagged cell surface receptors were initially immunolabelled prior to stimulation with U46619 for 30 min; thereafter, cells were permeabilized and HA-tagged receptor or GFP-tagged β-arrestin expression and co-localizations were captured by fluorescence microscopy (Fig. 8A and B). While TPβ, TPβ^C347S^ and TPβ^C347,373,377S^ were each clearly expressed on the cell surface in vehicle-treated cells (Fig. 8A and B), TPβ was significantly internalized even following exposure of cells to U46619 for 30 min in the presence of both GFP-tagged β-arrestin1 or β-arrestin2 (Fig. 8Ai and Bi). Moreover in the presence of either GFP-tagged β-arrestin1 or β-arrestin2, TPβ^C347S^ also underwent significant U46619-induced internalization (Fig. 8Aii and Bii). On the other hand, neither TPβ^C373S^, TPβ^C373,377S^ (data not shown) nor TPβ^C347,373,377S^ underwent internalization even in the presence of over-expressed β-arrestins (Fig. 8Aiii and Biii). Consistent with previous reports [50], in vehicle-treated cells, both β-arrestins exhibit diffuse uniform staining in the cytoplasm whilst β-arrestin1, but not β-arrestin2, also showed significant nuclear staining (Fig. 8A and B). Upon U46619-stimulation and in the presence of TPβ, the GFP staining pattern associated with both β-arrestin1 and β-arrestin2 was significantly altered. Specifically, GFP fluorescence became punctuate with a translocation of both β-arrestin1 and β-arrestin2 from the cytoplasm to the periphery of the cell, consistent with their co-localization and interaction with agonist-activated TPβ on the cell surface (Fig. 8Ai and Bi, respectively). Similarly, in the presence of TPβ^C347S^, both β-arrestin1 and β-arrestin2 showed translocation and enhanced co-localization with TPβ^C347S^ at the cell surface in response to U46619-stimulation (Fig. 8Aii and Bii). On the other hand, there was no evidence of β-arrestin1/2 translocation or receptor co-localization with TPβ^C373S^, TPβ^C373,377S^ (data not shown) or TPβ^C347,373,377S^ (Fig. 8Aiii and Biii) in response to U46619-stimulation. Taken together, these data demonstrated that over-expression of β-arrestin1 or β-arrestin2 could compensate for the defect in agonist-induced internalization of TPβ^C347S^ and suggest that palmitoylation of TPβ, in particular at Cys^347^, is specifically required to mediate β-arrestin1/2 translocation and receptor interaction in response to agonist-stimulation.

## 4 Discussion

Palmitoylation is a prevalent feature of GPCRs with up to 80% of the superfamily estimated to contain at least one cysteine in their C-tail domain, typically but not exclusively proximal to transmembrane (TM) 7, which may potentially be palmitoylated [5,7]. In this study, we sought to establish whether TPα and/or TPβ are palmitoylated and to investigate the functional consequences thereof. Whilst TPα does not contain any cysteines within its unique C-tail domain, TPβ possesses Cys^347^ close to the TM7/C-tail boundary and the couplet Cys^373^ and Cys^377^ located some 30 amino acids from its C-terminus. The results herein confirm that TPβ, but not TPα, is palmitoylated. Furthermore, site-directed mutagenesis and *in vivo* metabolic labelling determined that TPβ is palmitoylated at Cys^347^ and, to a lesser extent, at Cys^373,377^. Whilst palmitoylation was confirmed to occur at Cys^373^, attempts to further ascertain whether Cys^377^, along with Cys^373^, is specifically palmitoylated were not possible as stable cell lines over-expressing TPβ^C377S^ could not be generated despite repeated attempts.

A typical feature of palmitoylation is that it is viewed as a reversible modification, with the propensity to regulate protein:membrane or protein:protein association, protein localization and/or function in a dynamic manner [1,6]. Moreover, several GPCRs are known to undergo increased palmitoylation or depalmitoylation in an agonist-dependent manner, modulating receptor signalling potential in response to ligand engagement [13,51,52]. In the current study, we found no evidence of altered palmitoylation status of TPβ in response to agonist-stimulation under the conditions of metabolic labelling employed and, hence, data presented throughout are those generated in the absence of ligand activation. However, as the process of metabolic labelling and detection of palmitoylation is a relatively inefficient process, with poor-signal-to-noise ratio, we cannot exclude the possibility that TPβ palmitoylation is also subject to dynamic modulation, such as in response to agonist-engagement.

Numerous studies have indicated that the consequences of palmitoylation of GPCRs are wide-ranging with many aspects of receptor function being potentially implicated including receptor processing, G protein/effector coupling, phosphorylation and desensitization, sequestration and internalization, trafficking and/or protein turnover [1–5]. In examining the functional consequences herein it was established that palmitoylation of TPβ at Cys^347^ is required for its efficient Gq-mediated PLCβ activation, the primary effector of TP signalling. Specifically in the case in TPβ^C347S^ and TPβ^C347,373,377S^, there was a 30% reduction in U46619-mediated [Ca^2+^]_*i*_ mobilization compared to that of the wild type TPβ. In contrast, U46619-mediated [Ca^2+^]_*i*_ mobilization by TPβ^C373S^ and TPβ^C373,377S^ was not significantly different from TPβ, indicating that palmitoylation at Cys^373^ and/or Cys^377^ does not influence Gq/PLCβ effector signalling. The reduction in [Ca^2+^]_*i*_ mobilization by TPβ^C347S^ or TPβ^C347,373,377S^ was not due differences in their ligand binding properties as each of the palmitoylation deficient mutants displayed similar affinities for the selective TP radioligand [^3^H]SQ29,548 and their overall level of expression was not significantly different to that of the wild type TPβ. The specific requirement for palmitoylation at Cys^347^ for TPβ-mediated Gq/PLCβ activation is somewhat similar to that previously reported for the hIP where it was established that palmitoylation at Cys^308^, but not at Cys^311^, is specifically required for its Gq/PLCβ coupling [15].

A number of studies have demonstrated that impairment of receptor palmitoylation, as exemplified by the CCR5 receptor, can result in the accumulation of GPCRs in intracellular stores and in the lowering of cell surface expression [53]. In the case of TPβ and its variants, as stated, we have established that impairment of palmitoylation *per se* does not affect the overall level of surface expression and does not appear to affect the maturation or surface expression of TPβ. As TPβ is reported to undergo agonist-dependent and agonist-independent or tonic internalization, albeit through distinct mechanisms, we next sought to determine whether palmitoylation may affect TPβ internalization [34,35]. It was established that mutation of Cys^347^, Cys^373^ or Cys^377^, either alone or in combination, significantly impaired agonist-induced TPβ internalization reducing the movement of the respective cell surface receptors (TPβ^C347S^, TPβ^C373S^, TPβ^C373,377S^ or TPβ^C347,373,377S^) into intracellular compartment(s) relative to the wild type TPβ. On the other hand, while tonic internalization by TPβ^C373S^, TPβ^C373,377S^ and TPβ^C347,373,377S^ was also significantly impaired, it was striking that the variant TPβ^C347S^ retained the ability to undergo tonic internalization. Furthermore, over-expression of either β-arrestin1 or β-arrestin2 was able to overcome the inability of TPβ^C347S^ to undergo agonist-induced internalization, an effect not observed with the other palmitoylation deficient mutants including TPβ^C373S^, TPβ^C373,377S^ and TPβ^C347,373,377S^. These data indicated a critical role for palmitoylation of Cys^373,377^ in both agonist-dependent and tonic internalization of TPβ, while palmitoylation of TPβ^C347^ is also required for agonist-induced, but not tonic internalization.

Taken together, our results indicate that TPβ is palmitoylated at 2 independent sites Cys^347^ and Cys^373,377^ separated by some 30 amino acid residues within its unique C-tail domain, to regulate G protein coupling and receptor internalization. One of the structural consequences of GPCR palmitoylation is that, by increasing local hydrophobicity, the palmitoyl moiety(s) is thought to become inserted into the membrane lipid bilayer resulting in the formation of a putative fourth intracellular loop within its C-tail domain, such as originally demonstrated for rhodopsin [8,54,55]. Moreover, in certain cases, palmitoylation at multiple independent sites, either alone or in combination with other post-translational lipid modifications, has been proposed to result in the formation of loop structures within the C-tail domain of the given GPCR. For example, in the case of the 5-hydroxytryptamine (5-HT)_4(a)_ receptor, Ponimaskin et al. [13,48] established that it undergoes palmitoylation at two adjacent Cys residues (Cys^328^/Cys^329^) located proximal to TM7 but is also palmitoylated at Cys^386^, located one amino acid residue from the C-terminus. They proposed that membrane insertion of the palmitates at the proximal Cys^328^/Cys^329^ and distal Cys^386^ residues resulted in the formation of a double loop structure within the C-tail of the 5-HT_4(a)_ receptor. While palmitoylation deficient mutants of 5-HT_4(a)_ receptor were indistinguishable from the wild type receptor in mediating G protein/effector signalling, mutation of the proximal and distal residues was shown not only to differentially modulate constitutive activation but also to modulate agonist-dependent phosphorylation, desensitization and β-arrestin internalization of the 5-HT_4(a)_ receptor [13,48,56]. Similarly, it has been proposed that the C-tail domain of the hIP also contains a double loop structure anchored by dynamically regulated proximal palmitoyl groups, at Cys^308^/Cys^311^, and by a distal farnesyl isoprenoid permanently attached to its C-terminus and that collectively these dual lipidations modulate G protein coupling and effector signalling [15].

**Fig. 9:**
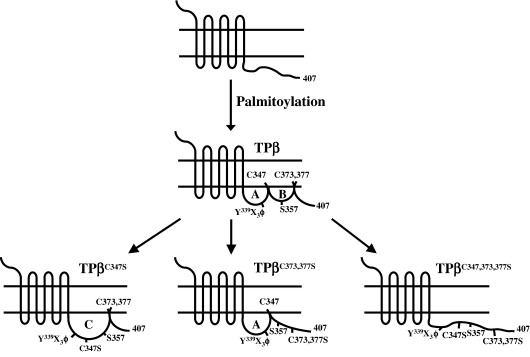
Model of palmitoylation of TPβ. Palmitoylation of TPβ occurs at 2 distinct sites through the attachment of palmitates at Cys^347^ and Cys^373/377^. Following palmitoylation, it is proposed that membrane insertion of the palmitate moieties at Cys^347^ and Cys^373/377^ leads to the formation of a double loop structure within the C-tail domain of TPβ referred to as Loop A and Loop B, respectively. A third conformation of the C-tail of TPβ occurs when Cys^373,377^, but not Cys^347^, is palmitoylated resulting in the formation of Loop C, such as is found in TPβ^C347S^. TPβ^C373,377S^ undergoes palmitoylation at Cys^347^ resulting in the formation of Loop A only while TPβ^C347,373,377S^ does not contain Loops A, B or C. It was found that Loop A is required for efficient Gα_q_/PLCβ coupling as evidenced by the reduced agonist-induced [Ca^2+^]_*i*_ mobilization by TPβ^C347S^ and TPβ^C347,373,377S^. It is proposed that Loop B is required for agonist-induced GRK phosphorylation at Ser^357^, followed by β-arrestin recruitment and TPβ internalization, as evidenced by the impaired U46619-induced β-arrestin1/2 recruitment and internalization by TPβ^C347S^, TPβ^C373,377S^ and TPβ^C347,373,377S^. Like the wild type TPβ, TPβ^C347S^ (which contains Loop C only) retains the ability to undergo tonic internalization while TPβ^C373,377S^ (which contains Loop A only) or TPβ^C347,373,377S^ fails to undergo tonic or agonist-induced internalization. From these latter data, it is proposed that palmitoylation at Cys^373^ and/or Cys^377^, resulting in the formation of Loop C only (in TPβ^C347S^) or Loop A plus Loop B (in wild type TPβ) is necessary to present a tonic internalization motif, such as the proposed Y^339^X_3_Φ^343^[35], in the correct orientation for interaction with the internalization machinery.

In order to rationalise the functional consequences of palmitoylation of TPβ we present a model, as outlined in Fig. 9, whereby it is proposed that palmitoylation of TPβ at Cys^347^ and at Cys^373,377^ also leads to the formation of a double loop structure within its C-tail domain. In our model, palmitoylation at Cys^347^ and membrane insertion leads to the formation of Loop A which, though not obligatory for Gq coupling, leads to more efficient Gq/PLCβ activation and [Ca^2+^]_*i*_ mobilization. On the other hand, palmitoylation at the couplet Cys^373,377^, leads to the formation of Loop B or C, depending on whether Cys^347^ is also palmitoylated or not, respectively (Fig. 9). Whilst palmitoylation at Cys^373,377^ is essential for tonic internalization, palmitoylation at both Cys^347^ and Cys^373,377^, leading to the formation of Loops A and B, is required for agonist-induced internalization. In investigating the mechanism of agonist-induced internalization of TPβ, Parent et al., established that amino acid residues between 355 and 366 within its unique C-tail are essential for such β-arrestin-dependent internalization [34]. Moreover, it has since been established that Ser^357^ within the latter internalization domain is a specific target for agonist-dependent G protein-coupled receptor kinase (GRK)2/3 phosphorylation leading to β-arrestin recruitment and TPβ desensitization and internalization [57]. It is indeed noteworthy that both the latter agonist-dependent internalization motif and the GRK phospho-target are located between the palmitoylation sites at Cys^347^ and Cys^373,377^ within the C-tail of TPβ. Hence in our model (Fig. 9) and as supported by our experimental findings, we propose that palmitoylation of TPβ at both Cys^347^ and Cys^373,377^, leading to the formation of Loops A and B, would expose the agonist-induced internalization motif and GRK phosphorylation site at Ser^357^ thereby enabling β-arrestin recruitment. Whilst formation of Loop C by palmitoylation at Cys^373,377^ alone, as observed in TPβ^C347S^, may also expose the internalization motif and GRK site at Ser^357^, we propose that the C-tail is in an altered, less favorable conformation for β-arrestin interaction and with reduced affinity for β-arrestin binding. Hence, palmitoylation of Cys^373,377^ alone, as in the case of TPβ^C347S^, yields a receptor with impaired agonist-induced internalization owing to its reduced affinity for β-arrestin1/2. Consistent with this, through our experimental observations, over-expression of either β-arrestin1/2 overcame this reduced affinity by TPβ^C347S^ and facilitated its internalization in response to U46619-stimulation to levels that were indistinguishable from those of the wild type TPβ. Moreover, our immunolocalization data supported the agonist-dependent recruitment of GFP-tagged forms of β-arrestin1 and β-arrestin2 to both the agonist-activated TPβ and TPβ^C347S^ but not to TPβ^C347,373,377S^, for example.

Our proposed model accounting for the role of palmitoylation in the regulation of β-arrestin-dependent agonist-induced internalization of TPβ is consistent with a number of other studies. In the case of the V2 vasopressin receptor, for example, Charest and Bouvier established that palmitoylation enhances the β-arrestin recruitment to the activated V2R, facilitating internalization and other processes requiring the scaffolding action of β-arrestin including activation of the mitogen-activated protein kinase cascades [16]. In contrast, other studies demonstrated that palmitoylation may actually inhibit β-arrestin interaction [58]. For example, palmitoylation of the luteinizing hormone/human chorionic gonadotropin receptor physically hinders receptor:β-arrestin interaction such that the palmitoylation status of the receptor determines whether internalization actually proceeds through a β-arrestin-dependent or independent mechanism [58].

Whilst TPβ undergoes agonist-induced internalization via a dynamin-, GRK2/3- and β-arrestin-dependent mechanism, requiring an active or dynamic actin cytoskeleton [34–36], tonic internalization is reported to occur, at least in part, through a dynamin-dependent mechanism with no requirement for either GRK phosphorylation or the β-arrestins [35]. In studies investigating the determinants of tonic internalization, Parent et al., identified the presence of a tonic internalization motif Y^339^X_3_Φ^343^, where X is any residue and Φ is a bulky hydrophobic isoleucine, within the C-tail domain of TPβ. Hence, the critical residues required for tonic internalization lie between residues 339 and 343 in close proximity to the Cys^347^ within the proposed Loop A structure associated with TPβ (Fig. 9). Hence, it is tempting to speculate that palmitoylation at Cys^347^ may modulate tonic internalization of TPβ. Our experimental finding that TPβ^C347S^, in addition to the wild type TPβ, undergoes tonic internalization suggests that the conformation of Loop C is sufficient to present or orientate the latter motif for internalization to occur. However, the fact that TPβ^C373,377S^ which contains Loop A, but not Loop B, does not undergo tonic internalization leads us to propose that other unidentified sequence determinants within Loop B, associated with TPβ, may also be required for efficient tonic internalization and that the conformation of Loop C may also orientate those sequences to facilitate that internalization. The identities of those additional sequences required for tonic internalization remain to be established.

In summary, various studies have indicated that many GPCRs are palmitoylated and that the functional consequences are diverse between receptors [3–5]. In this study we have established that TPβ, but not TPα, is palmitoylated and that palmitoylation occurs at distinct 2 sites, Cys^347^ and Cys^373,377^, within its unique C-tail domain. Whilst palmitoylation at Cys^347^ is required for efficient Gq/PLCβ effector coupling, palmitoylation at Cys^373,377^ is essential for receptor internalization, with palmitoylation at Cys^347^ acting as a structural determinant of the route by which the receptor is internalized, be it β-arrestin-dependent or independent internalization. Due to the importance of TXA_2_ in vascular hemostasis, a clearer understanding of how the TP isoforms are differentially regulated is critical. Palmitoylation provides a dynamic and reversible post-translational modification that can regulate TPβ, in terms of its signalling, receptor desensitization and internalization and, as such, acts as a major determinant of TPβ function. Moreover, data presented herein provide a further rational explanation for the clear functional and mechanistic differences between the TPα and TPβ isoforms.

## Figures and Tables

**Table 1:** Radioligand binding assays

Cell lines	*K*_d_	*B*_max_
	nM ± S.E.M.	pmol/mg protein ± S.E.M.
HEK.TPβ	8.44 ± 1.44	3.24 ± 0.33
HEK.TPβ^C347S^	7.81 ± 3.22	4.16 ± 0.86
HEK.TPβ^C373S^	8.21 ± 3.10	4.93 ± 1.05
HEK.TPβ^C373,377S^	8.57 ± 2.44	4.16 ± 2.06
HEK.TPβ^C347,373,377S^	8.00 ± 1.41	4.60 ± 0.30

Saturation radioligand binding isotherms were carried out on HEK 293 cells stably over-expressing HA epitope-tagged forms of TPβ and its variants using the TP antagonist [^3^H] SQ29,548 (50.4 Ci/mmol, 0–40 nM) and 75 μg of whole cell protein/assay. Radioligand binding data were analysed using GraphPrism 3 (GraphPad Software Inc.) to determine the *K*_d_ and *B*_max_ values. Data presented are the mean ± S.E.M. of 3 independent experiments.
