# Corrigendum to “Palmitoylation of the TPβ isoform of the human thromboxane A_2_ receptor. Modulation of G protein: Effector coupling and modes of receptor internalization.” [Cell Signal. 19(5) (2007) 1056–1070]

**DOI:** 10.1016/j.cellsig.2015.11.015

**Published:** 2016-03

**Authors:** Helen M. Reid, B. Therese Kinsella

**Affiliations:** School of Biomolecular and Biomedical Sciences, Conway Institute of Biomolecular and Biomedical Research, University College Dublin, Belfield, Dublin 4, Ireland

The authors regret that due to an error when preparing the panels, the incorrect data was presented in lane 1 of Panel B. The revised Fig. 1 showing the corrected Panel B is given below.

**Fig. 1:**
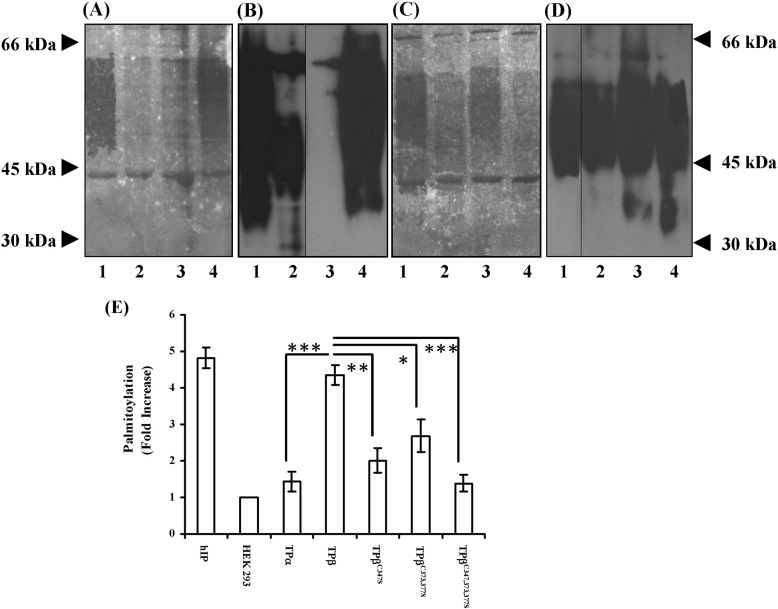
Analysis of palmitoylation in HEK.TPα and HEK.TPβ cells. Panels A and B, HEK.TPβ (lane 1), HEK.TPα (lane 2), HEK 293 (lane 3, negative control) and HEK.hIP (lane 4, positive control) cells were metabolically labelled for 2 h at 37 °C (Panel A). Panels C and D, HEK.TPβ (lane 1), HEK.TPβ^C347S^ (lane 2), HEK.TPβ^C373,377S^ cells (lane 3) and HEK.TPβ^C347,373,377S^ (lane 4) cells were metabolically labelled for 2 h at 37 °C (Panel C). Thereafter, the HA-tagged receptors were immunoprecipitated with anti-HA 101R and resolved by SDS-PAGE followed by electroblotting onto PVDF membrane. The blots (A and C) were soaked in Amplify prior to fluorography for 60–90 days at − 70 °C. Following fluorographic exposure, PVDF membranes, or parallel membranes, in A and C were screened by immunoblot analysis using anti-HA 3F10 peroxidase-conjugated antibody followed by chemiluminescent detection to obtain Panels B and D, respectively. The positions of the molecular weight markers (kDa) are indicated to the left and right of Panels A and D, respectively. Panel E, the level of palmitoylation in HEK.hIP, HEK.TPα, HEK.TPβ, HEKHA.TPβ^C347S^, HEK.TPβ^C373,377S^ and HEK.TPβ^C347,373,377S^ cells relative to basal levels, in HEK 293 cells, was determined by Phosphorimage analysis. Data is presented as mean fold increase in palmitoylation over basal levels ± S.E.M. and are expressed in arbitrary units. The data are representative of three independent experiments. The asterisks indicate that palmitoylation levels of TPα, TPβ^C347S^, TPβ^C373,377S^ and TPβ^C347,373,377S^ cells were significantly lower than that of TPβ where *p < 0.05; **p < 0.01; ***p < 0.005.

The authors would like to apologise for any inconvenience caused.

